# Effects of Egg White Protein Supplementation on Muscle Strength and Serum Free Amino Acid Concentrations

**DOI:** 10.3390/nu4101504

**Published:** 2012-10-19

**Authors:** Azumi Hida, Yuko Hasegawa, Yuko Mekata, Mika Usuda, Yasunobu Masuda, Hitoshi Kawano, Yukari Kawano

**Affiliations:** 1 Department of Nutritional Science, Faculty of Applied Bio-Science, Tokyo University of Agriculture, 1-1-1 Sakuragaoka, Setagaya, Tokyo 156-8502, Japan; Email: a3hida@nodai.ac.jp (A.H.); tenzo.hasegawa@nifty.com (Y.H.); 2 Faculty of Health and Nutrition, Bunkyo University, 1100 Gyouya, Chigasaki-City, Kanagawa 253-8550, Japan; Email: ymekata@shonan.bunkyo.ac.jp; 3 Institute of Technology R&D Division, Kewpie Corporation, 5-13-1 Sumiyoshi-cho, Fuchu-City, Tokyo 183-0034, Japan; Email: mika_usuda@kewpie.co.jp (M.U.); yasunobu_masuda@kewpie.co.jp (Y.M.); 4 Tokyo Metropolitan Institute for Neuroscience, 2-1-6 Kamikitazawa, Setagaya, Tokyo 156-8506, Japan; Email: kawano-ht@igakuken.or.jp

**Keywords:** egg white protein, muscle strength, female athletes, serum free amino acid, citrulline, urea

## Abstract

The aim of this study was to evaluate the effects of egg white protein compared to carbohydrate intake prior to exercise on fat free mass (FFM), one repetition maximum (1RM) muscle strength and blood biochemistry in female athletes. Thirty healthy female collegiate athletes were recruited for this study and matched by sport type, body fat percentage and 1RM leg curl muscle strength. Participants were randomly divided into two groups: protein group (15.0 g egg white protein; 75 kcal) and carbohydrate group (17.5 g maltodextrin, 78 kcal). Supplements were administered daily at the same time in a double-blind manner prior to training during an 8-week period. Measurements were performed before and after the 8-week regimen. The mean dietary energy intake did not change throughout the study period. FFM and 1RM assessments (*i.e.*, leg curl, leg extension, squat, and bench press) increased in both groups. Furthermore, serum urea and serum citrulline levels after the 8-week regimen increased significantly only in the protein group. Our findings indicated that compared to the carbohydrate supplement, the protein supplement was associated with some changes in protein metabolites but not with changes in body composition or muscle strength.

## 1. Introduction

Protein intake is an important component of body building, and together with additional supplements (*i.e.*, creatine and amino acids), is highly recommended for regular strength training. Male collegiate athletes consume more protein than what is recommended by the American Dietetic Association [1]. Bianco *et al.* reported that 30.1% of male athletes use dietary supplements during training as a “way to gain muscle and strength”, and also showed that whey protein shakes (50.0%) supplemented with creatine and amino acids (48.3%) were the most frequent choices amongst users [2]. Several reports describe how whey protein and amino acid supplementation increases muscle protein synthesis at rest [3,4] and after resistance training (RT) [5]. However, these studies were conducted in men. 

Josse *et al.* [6] evaluated the effects of milk and carbohydrate consumption during whole body RT in healthy women and whether or not such intake resulted in greater muscle mass accretion and muscle strength. They concluded that milk supplementation during RT effectively promoted changes in body composition. Nevertheless, there is a lack of reports in the field that explore such effects in female athletes. 

Depending on the quality of protein, it is well accepted that whey, casein, milk products and soy are dietary protein sources that are available to help increase fat free mass (FFM) or muscle strength performance [7,8,9]. In addition, supplementation of essential amino acids (EAA) together with normal protein intake has been shown to enhance protein accretion [10]. However, no detailed information on the effect of egg white protein intake is available, even though eggs are one of the cheapest animal protein sources in Japan, with the highest nutritional content [11]. Fifteen grams of egg white protein contain 1341 mg of leucine (Leu), 837 mg of isoleucine (Ile), and 1096 mg of valine (Val), and there is also an abundant source of branched amino acids (BCAA) and aromatic amino acids (AAA). Recent data showed that Leu induces a maximal skeletal muscle protein anabolic response in young adults [12], which suggests that Leu-rich egg white protein intake might have an important effect on body mass accretion.

The aim of this study was to determine the effect of egg white protein supplementation on body mass accretion, muscle strength performance and postprandial serum free amino acid concentration in female athletes. Measurements were taken before and after eight weeks of egg white protein (Prot group) or carbohydrate intake (Carb group). 

## 2. Experimental Section

### 2.1. Participants

Thirty college female athletes between the ages of 18 and 22 were recruited from the Japan Women’s College of Physical Education. All were well-trained athletes who engaged in regular training (e.g., volleyball and basketball) at least six times a week. Participants were asked to complete a medical screening questionnaire to identify any medical conditions prior to participation. All participants were deemed healthy and able to participate in the study. Participants were initially matched by age, sport type, body fat percentage (BF) and one repetition maximum (1RM) leg curl muscle strength. Participants were then randomly assigned to either an egg white protein supplement (Prot group, *n* = 15) or carbohydrate supplement (Carb, *n* = 15) group. The initiation of study tests was coordinated with each participant’s menstrual cycle (follicular phase). 

The importance of maintaining normal diet throughout the study was thoroughly explained, and participants were asked to maintain their baseline dietary habits. Furthermore, participants were not permitted to use any additional nutritional supplements, anabolic steroids, or other anabolic agents known to increase performance during the 8-week period and three months prior to initiation of the study. Participants with a history of egg protein allergy were excluded. All subjects were informed about the nature and possible risks associated with the experimental procedure, and written informed consent was obtained. The experimental protocol was approved by the Human Research Ethics Committee of Tokyo University of Agriculture. 

### 2.2. Study Design

The study design was a double-blind randomized concurrent trial test. Anthropometrical analysis, determination of daily energy expenditure and dietary nutrient intake and blood sample analysis were performed before (baseline) and after the 8-week supplementation regimen. Strength tests were conducted two days after blood drawings.

### 2.3. Protein and Carbohydrate Supplements

Protein supplements consisted of 15.0 g of dried egg white protein (75.0 kcal energy) and carbohydrate supplements consisted of 17.5 g of maltodextrin (78.0 kcal energy), with chocolate flavor included as the only additive (Table 1). These supplements were prepared isoenergetically and kindly provided by Kewpie Corporation, Tokyo, Japan. Each supplement was delivered as a dry powder in sealed packages and with a number code to ensure study blinding. Supplements were reconstituted in 200 mL of mineral water prior to intake, but otherwise stored in a refrigerator until use. Each participant consumed the same supplements during the 8-week period, and their adherence to the regimen was monitored daily (mean adherence, 99.8%). 

**Table 1:** Supplement compositions. Participants took 20 g/day of each supplement during the 8-week study period. Supplements were delivered as dry powder in sealed packages and identified by a number code to ensure study blinding. Supplements were stored in a refrigerator until use and reconstituted with 200 mL of mineral water prior to intake(20 g/Pack).

Group	Prot	Carb
Energy (kcal)	75	78
Protein (g)	15.0 ^#^	0.3
Fat (g)	0.8	0.8
Carbohydrate (g)	2.0	17.5 *
Water (g)	1.0	1.3
Ash (g)	1.3	0.1
Salt (mg)	0.7	0.0

^#^ Egg white protein; * maltodextrin.

### 2.4. Anthropometrical Analysis

Height and BW were measured while wearing light clothing and no shoes. Height was determined to the nearest 0.1 cm using a stadiometer (HM-20H, Uchida Co., Tokyo, Japan). Body weight (BW), BF, and FFM were determined to the nearest 0.1 kg and 0.1% using an electronic scale (BC-303 SV, Tanita Co., Tokyo, Japan). Thigh, calf and waist circumferences were measured to the nearest 0.1 cm three times, and the mean value was calculated. 

### 2.5. One Repetition Maximum Strength Tests

At baseline and 8 weeks after the regimen, participants performed 1RM strength tests by leg curl (LC), leg extension (LE), squat, and bench press exercises. Participants were familiarized with the exercise procedures one week prior to the start of the study and at week 7 during the study period. Proper lifting technique was demonstrated and practiced. The 1RM muscle strength exercises for LE and LC were conducted on machines developed specifically for LC and LE measurements (Senoh, Japan), and squat and bench press strength tests were conducted using free-weight and variable-resistance exercise machines (Senoh, Japan).

Maximum strength was estimated two days after blood was drawn according to previously published protocols [13]. Each participant was asked to perform a warm-up set three or four times using a resistance approximately 40%–60% of her perceived maximum. Following these warm-up sets, the weight load was changed to 95%–97% of the pre-estimated maximum strength and increased after each successful lift until failure was achieved. A repetition was considered valid only if the participant was able to complete the entire lift in a controlled manner without assistance. Three to 5-minute resting periods were introduced between lifts [14]. Bench-press tests were performed in a standard supine position. The squat exercise required the participant to rest an Olympic weightlifting bar across the trapezius muscle at a self-chosen location. The squat was then performed in a parallel position (closely monitored by study staff), which was achieved when the greater trochanter of the femur was lowered to the same level as the knee. Verbal encouragement was consistently provided during all 1RM attempts. None of the subjects experienced any joint pain or muscle soreness due to testing procedures.

### 2.6. Dietary Analysis and Estimated Energy Expenditure

Participants were asked to record their dietary intake three days before blood sample collection at baseline and after the 8-week study period. Dietary records were then reviewed and clarified in an interview with a registered dietitian and entered into a computer software system for analysis of nutrient composition using *Standard Tables of Food Composition in Japan* (5th edition) [15]. 

Daily energy expenditure was estimated by assessing physical activity levels according to the current recommended dietary reference intake for Japanese people [16]. Physical activity levels were thus estimated for three days in accordance to the dietary intake records. Participants reported in the questionnaire the time they got up and went to bed, the frequency and duration of high- and moderate-intensity activities, and walking and sedentary activities. Each activity was assigned a metabolic equivalent task (MET) value [17]. The number of hours spent on each activity was multiplied by its MET value, and all products were summed to give a total MET-hour score for that day. 

### 2.7. Blood Sample Analysis

Participants fasted for at least 12 h prior to sample blood collection. Venous blood samples were collected from an antecubital vein using a 21-gauge needle. A vacuum tube was used to collect blood for the analysis of glucose (BG), triglyceride (TG), albumin (Alb), insulin, cortisol, growth hormone (GH), myoglobin and free amino acid concentrations, and for the determination of creatine phosphokinase (CPK), aspartate aminotransferase (AST) and alanine aminotransferase (ALT) activity. To determine free amino acid (AA) content, serum aliquots were deproteinized with sulfosalicylic acid and analyzed on an automated amino acid analyzer (L-8500, Hitachi High-Technologies Co., Tokyo, Japan). Blood analyses were performed by Medical Laboratory Systems (Kanagawa, Japan), and samples for each participant were analyzed on the same run or assay plate. 

### 2.8. Statistics

Not all participants in the two groups attended the required post-test due to conflicts with college class schedules. Data from these participants were not included in the analysis. All parameters were expressed as mean ± standard error (SE). After assessing the distribution of all parameters, data were analyzed using a repeated two-way analysis of variance (ANOVA). All analyses were performed using SPSS version 19.0. *P* values <0.05 were considered statistically significant. 

## 3. Results

### 3.1. Subject Characteristics and One Repetition Maximum Strength Tests

The only time × group interaction effect in dietary intakes was for protein intake (higher in the protein group), but there were no interactions for energy or carbohydrate intake (Table 2). Daily energy and carbohydrate intakes remained unchanged in both groups, and the egg white protein supplementation resulted in significant increase in a daily protein intake in the Prot group up to 1.2 g/kg BW (*P* = 0.023). However, the dextrin intake did not bring such a change. 

**Table 2:** Participant characteristics. All values are expressed as mean ± SE. ANOVA, a repeated two-way analysis of variance. T × G, effect of time × group interaction. BW: body weight.

	Group	Baseline	After 8 weeks	ANOVA		
				Time	T × G	Group
Energy (kcal)	Prot ^(1)^	2147 ± 72	1997 ± 59	0.302	0.440	0.537
	Carb ^(2)^	1996 ± 55	1974 ± 42			
Protein (g/BW)	Prot	1.08 ± 0.07	1.23 ± 0.07 *^,#^	0.578	0.036	0.253
	Carb	1.08 ± 0.09	1.00 ± 0.07			
Carbohydrate (g/BW)	Prot	4.94 ± 0.35	4.65 ± 0.28	0.567	0.240	0.535
	Carb	4.48 ± 0.21	4.62 ± 0.18			
Height (cm)	Prot	165.1 ± 1.4	165.1 ± 1.4	0.457	0.573	0.683
	Carb	166.0 ± 1.6	166.1 ± 1.7			
Body weight (kg)	Prot	60.6 ± 1.9	60.0 ± 1.8	0.055	0.765	0.830
	Carb	61.1 ± 1.4	60.6 ± 1.4			
Body fat ratio (%)	Prot	23.2 ± 1.9	20.2 ± 1.2	<0.001	0.931	0.983
	Carb	23.2 ± 1.6	20.1 ± 1.2			
Fat free mass (kg)	Prot	46.3 ± 1.2	47.8 ± 1.1	0.004	0.958	0.708
	Car	46.8 ± 1.3	48.4 ± 1.1			
Thigh circumference (cm)	Prot	56.2 ± 0.9	56.4 ± 0.8	0.733	0.937	0.261
	Carb	54.8 ± 0.1	55.0 ± 0.1			
Calf circumference (cm)	Prot	37.6 ± 0.6	37.6 ± 0.5	0.714	0.440	0.722
	Carb	37.4 ± 0.5	37.2 ± 0.5			
Waist circumference (cm)	Prot	74.4 ± 1.5	75.4 ± 1.2	0.630	0.534	0.358
	Carb	76.7 ± 1.6	76.6 ± 1.2			
Leg extension (kg)	Prot	43.1 ± 2.0	48.2 ± 1.9	<0.001	0.756	0.194
	Carb	47.7 ± 2.4	52.7 ± 2.6			
Leg curl (kg)	Prot	39.2 ± 1.7	42.9 ± 1.7	<0.001	0.839	0.456
	Carb	42.1 ± 2.3	45.8 ± 2.2			
Squat (kg)	Prot	92.7 ± 3.8	99.5 ± 4.2	<0.001	0.486	0.129
	Carb	86.6 ± 3.8	96.5 ± 3.2			
Bench press (kg)	Prot	41.9 ± 2.8	44.1 ± 2.6	<0.001	0.581	0.100
	Carb	37.1 ± 1.5	40.4 ± 1.6			

^#^ Statistically significant by a non-paired *t*-test from the Carb group (*P* < 0.05); * Statistically significant by a paired *t*-test between baseline and after 8 weeks (*P* < 0.05); ^(^^1)^ an egg white protein supplement group; ^(^^2)^ a maltodextrin supplement group.

There was no significant difference between groups in regards to estimated daily energy expenditure during the study period. Participants in the Prot and Carb group consumed 2208 ± 96 kcal/day and 2201 ± 76 kcal/day at baseline or 2226 ± 58 kcal/day and 2200 ± 89 kcal/day after the 8-week regimen, respectively. Mean energy intakes tended to be lower than levels of energy expenditure.

No significant effect of the time × group interaction was observed, and a main effect of the groups was observed by each physical characteristics and 1RM strength (Table 2). In contrast, a main effect of the time in BF, FFM and all four strengths was significantly observed; BF was significantly lower, while FFM levels and all four 1RM strength tests were significantly higher in both groups after the 8-week regimen. Beside, height, BW, and thigh, calf and waist circumference did not change in both groups throughout the study period. 

### 3.2. Blood Sample Analysis

No significant interaction effect of the time × group in blood biochemistry was observed (Table 3). 

**Table 3:** Blood biochemistry. All values are expressed as mean ± SE. ANOVA, a repeated two-way analysis of variance. T × G, time × group interaction effect.

	Group	Baseline	After 8 weeks	ANOVA		
				Time	T × G	Group
Blood glucose (mg/dL)	Prot ^(1)^	74.6 ± 0.9	84.9 ± 1.8	<0.001	0.414	0.875
	Carb ^(2)^	76.1 ± 0.9	84.1 ± 1.8			
Triglyceride (mg/dL)	Prot	53.6 ± 6.1	67.0 ± 15.9	0.063	0.505	0.504
	Carb	55.6 ± 6.1	83.2 ± 15.9			
Albumin (g/dL)	Prot	4.7 ± 0.0	4.6 ± 0.0	0.022	0.103	0.597
	Carb	4.7 ± 0.1	4.7 ± 0.1			
Creatine phosphokinase activity (IU/L)	Prot	188 ± 20	153 ± 18	0.079	0.471	0.782
	Carb	194 ± 22	125 ± 14			
Aspartate aminotransferaseactivity (U/L)	Prot	21.9 ± 1.5	21.5 ± 1.3	0.079	0.601	0.218
	Carb	20.2 ± 1.3	18.7 ± 1.0			
Alanine aminotransferaseactivity (U/L)	Prot	17.0 ± 1.8	19.4 ± 2.4	0.940	0.109	0.006
	Carb	13.7 ± 1.2	12.2 ± 0.8			
Cortisol (μg/dL)	Prot	15.7 ± 0.8	18.6 ± 0.8	0.006	0.167	0.638
	Carb	17.1 ± 0.8	18.1 ± 1.1			
Insulin (μU/mL)	Prot	8.8 ± 0.2	8.5 ± 0.3	0.987	0.117	0.093
	Carb	9.5 ± 1.0	9.8 ± 0.6			
Growth Hormone (ng/mL)	Prot	6.1 ± 1.1	1.9 ± 0.3	0.001	0.645	0.756
	Carb	5.2 ± 0.7	2.5 ± 0.7			
Myoglobin (mg/dL)	Prot	56.8 ± 5.1	47.9 ± 4.1	0.001	0.565	0.726
	Carb	53.9 ± 3.5	43.5 ± 1.9			

^(1)^ An egg white protein supplement group; ^(2)^ a maltodextrin supplement group.

The main effect of the time was significant for BG, Alb, cortisol, GH and myoglobin concentrations. BG and serum cortisol concentrations were significantly higher at the 8-week compared to baseline measurement. Alb, GH and myoglobin concentrations decreased significantly at eight weeks. Furthermore, the main effect of the groups was observed only for ALT activities, which were significantly higher in the Prot compared to the Carb group. Serum TG, CPK and AST levels did not change throughout the study period in both groups.

### 3.3. Serum AA Concentrations and Protein Metabolites

No significant interaction effect of time × group in serum AA levels was observed (Table 4). The main effect of the time was significant for some AA concentrations of Val, Leu, Ile, lysine, Ala, asparagine, glutamic acid, tyrosine and proline, respectively (Table 4). The main effect of the groups was only observed in the tryptophan (Trp) concentrations, which were more significant in the Prot group compared to the Carb group. In addition, each concentration of total AA, total EAA, BCAA and AAA increased significantly after the 8-week regimen, respectively, and the Trp to BCAA ratio decreased significantly after 8 weeks.

**Table 4:** Changes in serum free amino acid concentrations. All values are expressed as mean ± SE. EAA, essential amino acid. NEAA, non essential amino acid. BCAA, branched chain amino acid. AAA, aromatic amino acid. ANOVA, a repeated two-way analysis of variance. T × G, time × group interaction effect.

		Group	Baseline	After 8 weeks		ANOVA		
						Time	T × G	Group
EAA	Valine (nmoles/mL)	Prot ^(1)^	201 ± 7	223 ± 7		0.004	0.976	0.914
		Carb ^(2)^	198 ± 8	222 ± 10				
	Leucine (nmoles/mL)	Prot	113 ± 4	129 ± 4		0.004	0.893	0.903
		Carb	115 ± 6	130 ± 8				
	Isoleucine (nmoles/mL)	Prot	60.4 ± 2.2	72.0 ± 2.8		0.002	0.516	0.874
		Carb	62.9 ± 3.0	71.6 ± 4.6				
	Threonine (nmoles/mL)	Prot	120 ± 9	128 ± 6		0.305	0.411	0.948
		Carb	122 ± 5	122 ± 5				
	Methionine (nmoles/mL)	Prot	33.2 ± 1.8	37.1 ± 1.3		0.357	0.220	0.472
		Carb	33.3 ± 1.3	33.9 ± 1.0				
	Phenylalanine (nmoles/mL)	Prot	63.5 ± 2.0	70.2 ± 2.1		0.123	0.134	0.303
		Carb	63.6 ± 2.3	64.6 ± 2.1				
	Lysine (nmoles/mL)	Prot	152 ± 6	171 ± 7		0.010	0.339	0.922
		Carb	155 ± 9	167 ± 7				
	Tryptophan (nmoles/mL)	Prot	61.3 ± 2.5	63.8 ± 2.7		0.385	0.911	0.029
		Carb	56.3 ± 2.3	58.2 ± 2.1				
	Histidine (nmoles/mL)	Prot	80.5 ± 2.0	84.3 ± 3.1		0.198	0.683	0.630
		Carb	77.9 ± 2.9	81.5 ± 3.0				
NEAA	Glycine (nmoles/mL)	Prot	236 ± 15		251 ± 12	0.235	0.421	0.841
		Carb	235 ± 10		237 ± 11			
	Alanine (nmoles/mL)	Prot	342 ± 26		392 ± 15	0.028	0.164	0.142
		Carb	387 ± 18		404 ± 16			
	Serine (nmoles/mL)	Prot	114 ± 4		118 ± 4	0.419	0.055	0.398
		Carb	126 ± 6		116 ± 6			
	Cysteine (nmoles/mL)	Prot	17.4 ± 0.9		18.4 ± 0.6	0.903	0.070	0.453
		Carb	18.6 ± 1.1		18.1 ± 1.0			
	Asparagine (nmoles/mL)	Prot	44.7 ± 1.2		51.5 ± 1.4	0.003	0.054	0.849
		Carb	47.2 ± 2.3		49.3 ± 2.0			
	Glutamic acid (nmoles/mL)	Prot	20.1 ± 1.1	26.9 ± 2.3		<0.001	0.909	0.786
		Carb	21.3 ± 1.2	26.8 ± 2.9				
	Glutamine (nmoles/mL)	Prot	543 ± 25	573 ± 15		0.521	0.073	0.884
		Carb	561 ± 14	549 ± 19				
	Arginine (nmoles/mL)	Prot	88.0 ± 4.1	101.5 ± 4.9		0.098	0.084	0.489
		Carb	98.5 ± 4.1	98.2 ± 5.0				
	Tyrosine (nmoles/mL)	Prot	62.2 ± 2.6	73.0 ± 3.1		0.004	0.063	0.487
		Carb	64.3 ± 1.9	66.8 ± 2.4				
	Proline (nmoles/mL)	Prot	132 ± 11	153 ± 12		0.002	0.832	0.991
		Carb	135 ± 14	160 ± 18				
Total AA (nmoles/mL)		Prot	2638 ± 86	2905 ± 70		0.009	0.228	0.895
		Carb	2731 ± 86	2835 ± 70				
Total EAA (nmoles/mL)		Prot	827 ± 28	924 ± 28		0.004	0.593	0.627
		Carb	826 ± 28	895 ± 28				
BCAA (nmoles/mL)		Prot	375 ± 15	424 ± 17		0.002	0.955	0.876
		Carb	376 ± 15	428 ± 17				
Trp/BCAA ratio (%)		Prot	16.5 ± 0.7	15.2 ± 0.7		0.014	0.940	0.074
		Carb	15.1 ± 0.6	13.8 ± 0.5				
AAA (nmoles/mL)		Prot	185 ± 13	207 ± 6		0.041	0.121	0.100
		Carb	127 ± 5	136 ± 5				

^(1)^ An egg white protein supplement group; ^(2)^ a maltodextrin supplement group.

**Figure 1:**
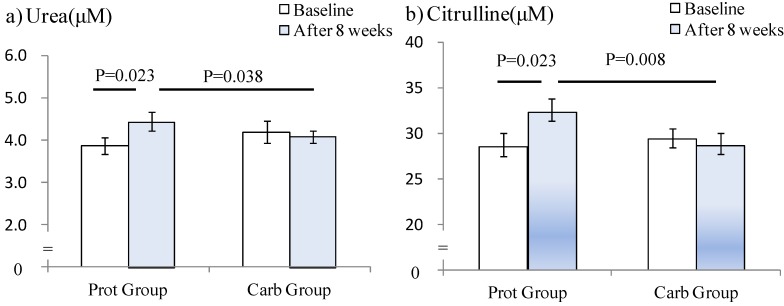
Changes in serum urea (**a**) and citrulline (**b**) concentrations. Data are expressed as mean ± SE. A time × group interaction effect was observed for both serum urea (*P* = 0.025) and serum citrulline concentrations (*P* = 0.029).

For protein metabolites, the time × group interaction effect was significant for serum urea (*P* = 0.025) or serum citrulline (*P* = 0.029) concentrations (Figure 1). Changes in serum urea or serum citrulline concentrations measured after the 8-week regimen were significantly higher in the Prot compared to the Carb group (*P* = 0.038, 0.008, respectively). Taurine concentrations increased significantly in both groups (main effect of the time, *P* = 0.002; data not shown), but the ornithine levels did not change in both groups throughout the study period.

## 4. Discussion

To our knowledge, this study is the first to investigate the effect of egg white protein supplementation on changes in FFM, 1RM strength and serum free AA in female athletes. Our findings showed that daily supplementation of either carbohydrate or protein resulted in increase in the FFM and 1RM muscle strength, and that the only separate effect of the protein supplementation was the increases in ALT activity, serum urea and serum citrulline concentrations. These observations suggested that a small increase in the energy intakes and the training during 8-week periods was related to increased FFM levels, muscular strength and some AA concentrations, and that daily protein supplementation up to 1.2 g/kg BW resulted in increase in the protein metabolites.

In our present study, daily protein intake in the Prot group was 1.08 g/kg BW and 1.23 g/kg BW at baseline and 8-week measurements, respectively. Furthermore, participant BW remained unchanged throughout the study while BF decreased significantly. Cooke *et al.* [18] examined the effects of whey protein intake (1.5 g/kg BW, ~30 g) during a 14-day period on protein levels and muscle force recovery after eccentrically-induced muscle damage in healthy individuals (80 ± 11 kg BW), and reported that whey protein supplementation attenuated muscle force impairment that occurs during recovery from exercise-induced muscle injury. In their study, participants consumed sources of energy (30 kcal/kg BW), protein (0.8 g/kg BW) and carbohydrate (4.5 g/kg BW) with an overall protein intake of 2.3 g/kg BW, suggesting that supplemental sources were needed to meet the levels recommended by the American Dietetic Association [18]. Josse *et al.* [6] examined the effect of milk protein supplementation on body composition in young healthy women (BW, 72.0 kg), and reported that milk supplementation during RT results in favorable changes to body composition, but that BW remained constant over a 12-week period. Participants in this previously published study consumed 70 g/day of dietary protein and 20 g/day of milk supplement, which resulted in a higher daily protein intake (0.97 g/kg BW to 1.24 g/kg BW). Changes in BW and BF observed in our study agree with those reported by Josse *et al.* [6]. In fact, participants in the two studies consumed nearly the same amount of dietary protein with (1.2 g/kg BW) or without (1.0 g/kg BW) protein supplements, suggesting that protein levels met the recommended standards [1]. 

In our present study, we set an intervention period for 8-weeks. Several studies have reported the efficacy of nutritional intake on muscle protein biosynthesis [19,20,21,22]. Fujita *et al.* [19] reported that EAA and carbohydrate intake prior to RT had no effect on muscle synthetic rates. Kerksick *et al.* [20] evaluated the effect of whey protein supplementation on muscle strength during a 10-week period of RT (48 g/day), and reported significant increases in 1RM bench press and leg press muscle strengths. In contrast, some studies have examined the effect of protein supplement timing on muscle strength and power in trained men during a 10-week period [21] and in healthy and physically active, yet untrained, male high school students during a 16-week period [8]. These studies revealed that training produces a significant increase in 1RM strength but no differences with the supplement regimen used [21]. On the other hand, Burk *et al.* [8] investigated the effect of casein-based supplement intake on the efficacy of RT during an 8-week period. Participants in this previously published study consumed 70 g of a protein supplement twice daily (*i.e.*, morning and five hours after training). The overall protein consumption (food plus supplement) was 2.2 g/kg BW. Burk *et al.* [8] reported that changes in BW and composition took place mainly during the first 8-week period, with a significant increase in 1RM muscle strengths. For this reason, the 8-week study period was chosen for our study. However, it is not clear if an 8-week period is sufficient to effectively evaluate BF loss. 

It is important to note that changes in body composition observed in our study were similar to those observed by Burk *et al.* [8]. We did observe that protein or carbohydrate supplementation in combination with daily training increases FFM levels [22], and results in higher 1RM strengths (although values seemed to be lower compared to those previously reported for males [23] or females [6]). Although we did not directly measure changes in lean muscle mass (assessed by dual energy X-ray absorptiometry), gains in muscle strength have been shown to be good surrogates for hypertrophy. FFM and 1RM strengths assessed in the present study increased during the 8-week period in both groups. However, it is unclear whether or not such gains arise from the energy supplementation, daily training, or both; it is not clear whether or not there is any relationship between small energy supplementation prior to daily training and muscle synthesis. Further studies are needed to clarify this point. 

Previous studies have evaluated changes in blood AA concentrations following a long-term energy intake and found that serum AA concentrations change temporarily during acute training [3,4,22] or energy intake [24]. Kesteloot *et al.* [25] studied the relationship between dietary protein intake and serum creatinine, urea and uric acid concentrations, and reported significant correlations in both sexes for total protein and animal and vegetable protein intake. Citrulline is an amino acid that is not involved in protein synthesis, but it restores nitrogen balance [26] and plays a pivotal role in maintaining protein homeostasis [27]. Likewise, urinary urea levels have been shown to increase significantly after RT [28], and serum and urinary taurine levels increase significantly during and after exercise [29]. These observations suggest that training increases myofibrillar or body protein breakdown. In our study, serum urea and serum citrulline increased significantly only in the Prot group after the 8-week regimen, and serum taurine increased in both groups. Therefore, our results suggest that protein supplementation increases protein metabolites (*i.e.*, urea and citrulline). In addition, serum ALT activity increased significantly only in the Prot group, even though it was within the normal range. These observations strongly suggested that an increase in daily protein intake up to 1.2 g/kg BW is related to increased protein metabolites. 

The present study showed that serum cortisol concentrations increase after the 8-week regimen. Cortisol increases hepatic lipolysis and proteolysis to fuel hepatic gluconeogenesis, which protects against changes in BG, increases TG levels and promotes muscle mass hypertrophy [30]. It is likely that the relatively lower energy intake compared to energy expenditure in our participants resulted in the observed increased serum cortisol concentrations, and thus it did not promote a lower BW. Serum CPK activity is sometimes used as a marker of muscle damage [31]. We also found that CPK levels remained unchanged, but muscle 1RM strengths were increased significantly in both groups, suggesting that protein or carbohydrate supplementation might inhibit muscle damage, which attenuates increased muscle strength. This in turn results in decreased myoglobin concentrations. 

There are a few limitations in this study that are worth noting. First, during this intervention period, the participants had a meal freely and did not establish a limit for the physical activity. Second, the study period was too short to distinguish the effects of protein and carbohydrate supplementation. Finally, the mechanism(s) involved in increased FFM, 1RM strength, serum, and some AA concentrations remain unknown. However, in our study, the most likely explanation for the improved FFM, 1RM strength and some of the free amino acids was that these well-trained female athletes continued training throughout the study period, suggesting that the training during 8-week regimen resulted to these increases. Furthermore, increased protein metabolites were only in the Prot group, suggesting that an increase in daily protein intake up to 1.2 g/kg BW is related to increased protein metabolites. Future studies are needed to further support our conclusions. 

## 5. Conclusions

Many male collegiate athletes consume more protein than what is recommended by the American Dietetic Association during regular training, and several reports describe that whey, casein, milk and soy protein are available to help increase FFM, or resistance muscle strength. In the present study, egg white protein supplementation caused a significant increase in the resistance muscle strength as well as carbohydrate supplementation in female athletes. Then, it seems likely that the training during the 8-week periods was related to enhancement of the muscular strength because a supplement of 75 kcals of either carbohydrate or protein increased FFM and strength. In addition, intakes of daily protein and increases in protein metabolite levels were only in the Prot group, suggesting that an increase in daily protein intake up to 1.2 g/kg BW resulted in an increase in the protein metabolites. Although the protein supplement compared to the carbohydrate supplement was associated with some changes in protein metabolites, there were no effects of the protein supplement on the body composition or strength measures. Thus, longer studies and/or higher protein intakes may be needed to observe the effects of these changes in protein metabolites on measures of body composition or strength. 
